# Ongoing burden of disease and mortality from HIV/CMV coinfection in Africa in the antiretroviral therapy era

**DOI:** 10.3389/fmicb.2015.01016

**Published:** 2015-09-24

**Authors:** Emily Adland, Paul Klenerman, Philip Goulder, Philippa C. Matthews

**Affiliations:** ^1^Department of Paediatrics, Peter Medawar Building for Pathogen Research, University of OxfordOxford, UK; ^2^Nuffield Department of Medicine, Peter Medawar Building for Pathogen Research, University of OxfordOxford, UK; ^3^Department of Infectious Diseases and Microbiology, John Radcliffe Hospital, Oxford University Hospitals NHS TrustOxford, UK; ^4^National Institute for Health Research Biomedical Research CentreOxford, UK; ^5^HIV Pathogenesis Programme, Doris Duke Medical Research Institute, University of KwaZulu-NatalDurban, South Africa

**Keywords:** HIV-1, CMV, coinfection, sub-Saharan Africa, pediatric infectious diseases, immune activation, antiretroviral therapy

## Abstract

Human Cytomegalovirus (CMV) is a well-recognized pathogen in the context of HIV infection, but since the roll out of ART, clinical and scientific interest in the problem of HIV/CMV coinfection has diminished. However, CMV remains a significant cofactor in HIV disease, with an influence on HIV acquisition, disease progression, morbidity, and mortality. Disease manifestations may be a result of direct interplay between the two viruses, or may arise as a secondary consequence of immune dysregulation and systemic inflammation. The problem is most relevant when the rates of coinfection are high, most notably in sub-Saharan Africa, and in children at risk of acquiring both infections early in life. Understanding the interplay between these viruses and developing strategies to diagnose, treat and prevent CMV should be a priority.

## Introduction

Human cytomegalovirus (CMV) is a ubiquitous β-herpesvirus, also known as Human Herpes Virus 5 (HHV5). It is the largest of the human herpesviruses with a 230 kb genome encoding 165 genes. CMV is widely recognized as an opportunistic pathogen, and has a high profile as an agent of disease in immunocompromised patients; much of the recent research literature addresses infection in those undergoing solid organ and bone marrow transplantation (Crough and Khanna, 2009; Gracia-Ahufinger et al., 2015; Itzykson et al., 2015; Stevens et al., 2015).

CMV disease in the context of HIV is equally well-established, although interest in the manifestations of coinfection has substantially diminished in the ART era. However, the complex interplay between these two chronic viral infections continues to be potentially highly significant, both in adults and children, and particularly in certain vulnerable populations, including sub-Saharan Africa, where both CMV and HIV are endemic in neonates and children, leading to a risk of coinfection *in utero* and during the earliest days of life (King et al., 2013). There has recently been a revival of interest in CMV as a cofactor for HIV infection and expert opinion has drawn attention to this somewhat neglected topic in the literature (Emery, 2014).

We have therefore set out to review the epidemiology, immunology and clinical impact of CMV infection in the setting of co-infection with HIV, focusing particularly on it's impact in southern African populations—especially in children who are at high risk of acquiring both infections within the first year of life.

We performed a PubMed search to identify reports of HIV/CMV coinfection, particularly representing studies conducted in sub-Saharan Africa. We have restricted references to focus primarily on publications from within the last 10 years. As there is a paucity of data from Africa, we have also cited studies of other populations where they raise points that are of particular relevance.

## Epidemiology of CMV mono- and co-infection

### Transmission and risk factors

CMV can be transmitted via saliva, sexual contact, placental transfer, breastfeeding, blood transfusion, and transplantation of solid-organs or haematopoietic stem cells. In African studies, factors including poor nutrition and low weight (Tembo et al., 2015), crowded living conditions (Alao et al., 2009) and other herpes virus coinfections (Schaftenaar et al., 2014) are associated with increased CMV seroprevalence. Maternal CMV viraemia during pregnancy is a risk factor for perinatal CMV transmission (Munro et al., 2005).

In the Western world, the rate of CMV seropositivity is much higher among adults with risk factors for acquisition of HIV infection, for example MSM (Gianella et al., 2015), than in the general population. Likewise, in a West African cohort, increased CMV seropositivity has also been demonstrated in HIV-positive individuals compared to HIV-uninfected controls (Compston et al., 2009). These observations all suggest shared risk factors for acquisition of the two viruses (e.g., lifestyle, behavior, demographics) as well as raising the possibility that HIV infection may be a direct risk factor for CMV acquisition in adults. The reciprocal scenario may also apply, as recent data from Africa suggest that CMV infection may be a risk factor for HIV transmission; a study in KwaZulu-Natal found an independent association between CMV in breast milk and postnatal mother-to-child transmission of HIV (Viljoen et al., 2015).

### Seroprevalence in adults and children

**Figure 1:**
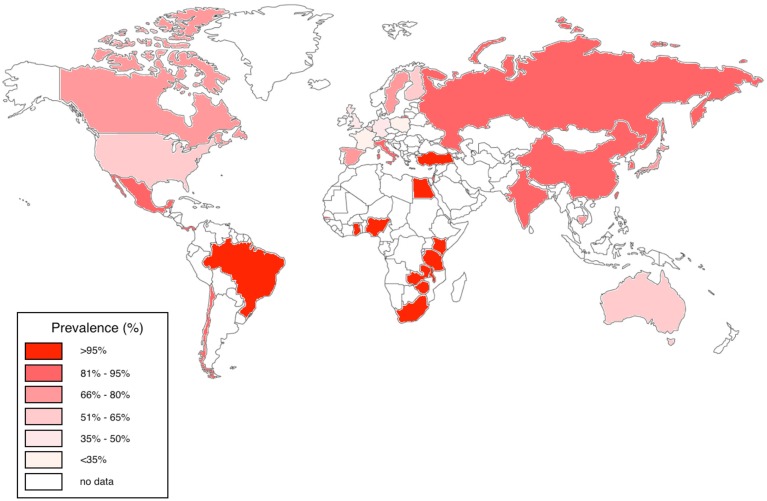
**Worldwide CMV seroprevalence rates in adults**. We have represented studies of adults aged 16–50 years published between 2005 and 2015 from Australia, Belgium, Brazil, Canada, Cambodia, Chile, China, Finland, France, Gambia, Germany, Ghana, India, Israel, Italy, Japan, Kenya, Mexico, Nigeria, Panama, South Africa, Spain, Sweden, Taiwan, Tanzania, Turkey, UK, USA, Zambia, and Zimbabwe (Chakraborty et al., 2003; Schlesinger et al., 2005; Miles et al., 2007, 2008; van der Sande et al., 2007; Zhang et al., 2007; Dar et al., 2008; Alao et al., 2009; Compston et al., 2009; Micol et al., 2009; Pass et al., 2009; Cannon et al., 2010; Chakravarti et al., 2010; Fielding et al., 2011; Brantsæter et al., 2012; Hsiao et al., 2013; Manicklal et al., 2013, 2014; Gumbo et al., 2014; Lanzieri et al., 2014; Mwaanza et al., 2014; Schaftenaar et al., 2014; Lichtner et al., 2015; Tembo et al., 2015; Viljoen et al., 2015).

In the USA, Australia and Europe, CMV seroprevalence among adults is variable, estimated at between 36 and 77%; in contrast, CMV is highly endemic in developing countries and in particular in sub-Saharan Africa, with a seropositivity rate that often approaches 100% in adults (Chakraborty et al., 2003; Schlesinger et al., 2005; Miles et al., 2007, 2008; van der Sande et al., 2007; Zhang et al., 2007; Dar et al., 2008; Alao et al., 2009; Compston et al., 2009; Micol et al., 2009; Pass et al., 2009; Cannon et al., 2010; Chakravarti et al., 2010; Fielding et al., 2011; Brantsæter et al., 2012; Hsiao et al., 2013; Manicklal et al., 2013, 2014; Gumbo et al., 2014; Lanzieri et al., 2014; Mwaanza et al., 2014; Schaftenaar et al., 2014; Lichtner et al., 2015; Tembo et al., 2015; Viljoen et al., 2015) (Figure 1).

Congenital CMV accounts for a significant burden of morbidity, including sensorineural deafness, developmental delay, and intrauterine growth retardation. This is estimated to affect 0.2–3% of live births in Western Europe and up to 14% in developing countries (Schlesinger et al., 2005; Zhang et al., 2007; Cannon et al., 2010; Manicklal et al., 2013, 2014; Gumbo et al., 2014; Mwaanza et al., 2014). The prevalence of congenital CMV in different African populations is likely to vary; data are sparse but studies range from rates of 2.9% in South Africa (Manicklal et al., 2014), to 3.8% in Zambia (Mwaanza et al., 2014), and 5.4% in the Gambia (van der Sande et al., 2007). Subsequently, almost two thirds of infants in African studies show serological evidence of infection by 3 months of age and 85% are infected by a year (Miles et al., 2007; Gumbo et al., 2014). By adolescence, CMV IgG is thought to be virtually universal in sub-Saharan African populations (Chakraborty et al., 2003).

In resource-poor settings, a large proportion of children undergo simultaneous primary HIV and CMV infection (Miles et al., 2007; Slyker et al., 2009). Thus, among HIV-positive children in Africa, the majority are coinfected with CMV by their first birthday (Miles et al., 2008; Gumbo et al., 2014), and almost all are coinfected by the time they reach their teens (Chakraborty et al., 2003). The growing epidemic of coinfected adolescents may have significant clinical impact in these populations. In comparison, a study from North America reports a lower—although still substantial—rate of early coinfections, finding that 40–50% of HIV/CMV-coinfected children had acquired CMV before their first birthday (Kovacs et al., 1999).

CMV infection is more common in neonates with HIV infection compared with HEU infants; to date it has not been possible to determine what proportion of CMV infections were acquired *in utero* (Tembo et al., 2015). Several mechanisms may explain the increased prevalence of CMV in HIV-infected neonates; first, HIV-infected mothers who transmitted HIV *in utero* would be expected to be more immunosuppressed and therefore at increased risk of reactivating and transmitting CMV. Second, immunosuppression associated with fetal HIV infection may result in higher risk of CMV acquisition *in utero*. Alternatively, fetal CMV coinfection may facilitate HIV acquisition *in utero* (Slyker et al., 2009).

## Immune control of CMV

**Figure 2:**
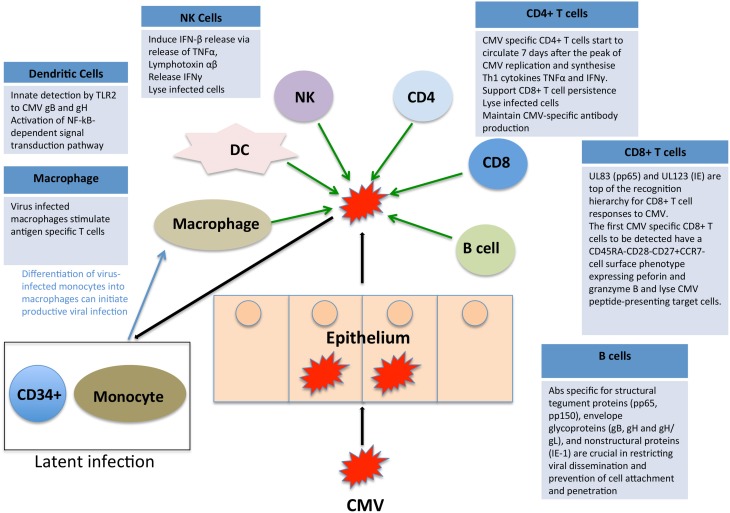
**Impact of the immune system on CMV**. CMV immune control is reliant on both the innate and adaptive arms of the immune system. We have here summarized the key elements, highlighting the complex interplay of multiple limbs of the immune system in containing CMV infection.

CMV immune control is multifactorial and there is evidence to suggest that CD8+ and CD4+ T cells, NK cells and antibody-mediated immunity all play a role in containment. We have summarized the key elements in Figure 2, highlighting the complex interplay of multiple limbs of the immune system in containing CMV infection. When any component of this immune control is aberrant, there is an increased risk of disease either due to primary CMV or reactivation of latent CMV, both of which can lead to disease manifestations.

There is also evidence that CMV can impact on immunological development, both in children and in adults (Ben-Smith et al., 2008; Miles et al., 2008a,b). In one study of adolescents, the CMV-positive group (mostly from Malawi) had fewer naïve CD4+ and CD8+ T cells and more differentiated memory T cells than CMV-negative subjects (Ben-Smith et al., 2008). This immunological phenotype is in keeping with that normally seen in older adults and equates with less robust T cell responses to new pathogens. Interestingly, CMV infection may also have a beneficial effect on immune responses to other pathogens: there are data from the Gambia suggesting that CMV infection can promote active T-cell responses in infancy, leading to more robust CD8+ responses to measles immunization and to staphylococcal enterotoxins (Miles et al., 2008).

## Immunopathology of HIV/CMV coinfection

One African study reports a strong correlation between peak HIV viral load and peak CMV viral load (Slyker et al., 2009). There are several possible explanations for this. First, a direct influence may operate between HIV and CMV viraemia, although it is impossible to infer causation or the direction of such a relationship: whether symptomatic CMV disease is a cause or effect of high HIV-p24 antigen concentration is not known. Second, it is also likely that there is an indirect effect, whereby systemic inflammation fuels the replication of both viruses (Griffiths, 2006). Finally, similar host factors may affect the containment of both viruses. Several direct influences of CMV on HIV have been proposed, as follows:

### Increased infection of CD4+ T cells by HIV

*In vitro* investigations suggest a synergistic effect of CMV and HIV in infecting CD4+ T cells and mediating immune suppression. CMV does not itself infect CD4+ T cells, but may enhance the uptake of HIV into these cells by several different mechanisms:

- The US28 gene of CMV encodes a chemokine receptor that can act as a substitute for the HIV receptor CCR5, so facilitating entry of HIV into CD4+ T cells (Pleskoff et al., 1997).
- CMV encodes a molecule which can act as an alternative receptor for HIV; HIV coated in non-neutralizing antibodies can gain access to fibroblasts via this CMV-encoded Fc receptor (McKeating et al., 1990). However, the significance of this process is uncertain, as fibroblasts do not support productive HIV infection.
- Active CMV infection can alter the cell tropism of HIV in dually infected individuals by facilitating HIV DNA entry into ordinarily non-permissive human fibroblasts (Margalith et al., 1995). For example, activation induced by CMV upregulates expression of CCR5 in CD4+ T central memory cells and facilitates *in utero* transmission of HIV (Johnson et al., 2014).

### Re-activation of latent HIV

CMV may activate latent proviral HIV DNA by several different mechanisms at the molecular level, briefly outlined below, and also explored by other reviews (Griffiths, 2006):

- The CMV IE-2 gene can augment HIV gene expression within the same cell (Davis et al., 1987).
- CMV infection causes release of cytokines from a bystander cell, activating latent HIV provirus through signal transduction (Clouse et al., 1989).
- Exposure to CMV antigen from a bystander cell could promote activation of latent HIV in a T memory cell whose cognate antigen receptor is CMV-specific (Peterson et al., 1992).
- Intermediate and early CMV genes can increase HIV expression by induction of NF-kappa B transcription factor, activating transcription from a stably expressed HIV-1 long terminal repeat (Davis et al., 1987).
- CMV upregulates cytokines including IL-2, IL-6, and TNFa that may contribute to inducing HIV replication (Osborn et al., 1989).

### Cellular immune activation and apoptosis

Cellular activation caused by CMV contributes to HIV pathogenesis by depletion of T cells via apoptosis induced cell death (AICD) (Slyker et al., 2012). Apoptosis is a hallmark of HIV infection and increased AICD is thought to contribute to the gradual loss of CD4+ helper cells during progression toward AIDS. The CD95/APO-1/Fas receptor/ligand system is critically involved in induction of apoptosis (Böhler et al., 2001; Cummins and Badley, 2010). Dysregulation of this system contributes to increased AICD of T cells in HIV infection and is associated with disease progression (Sloand et al., 1997; Silvestri et al., 1998; Böhler et al., 2001; Cummins and Badley, 2010).

In a cohort of Kenyan HIV-infected infants, the frequencies of activated (CD38+ HLA-DR+) and apoptosis vulnerable (CD95+Bcl-2-) CD4+ and CD8+ T cells increased substantially during acute CMV infection (Slyker et al., 2012). Similar observations were made in HEU infants (Slyker et al., 2012). These data support the hypothesis that CMV-induced T cell activation and Fas-mediated apoptosis potentially contribute to the increased HIV disease progression observed particularly in CMV coinfected infants (Kovacs et al., 1999).

## Clinical consequences of CMV/HIV coinfection

**Figure 3:**
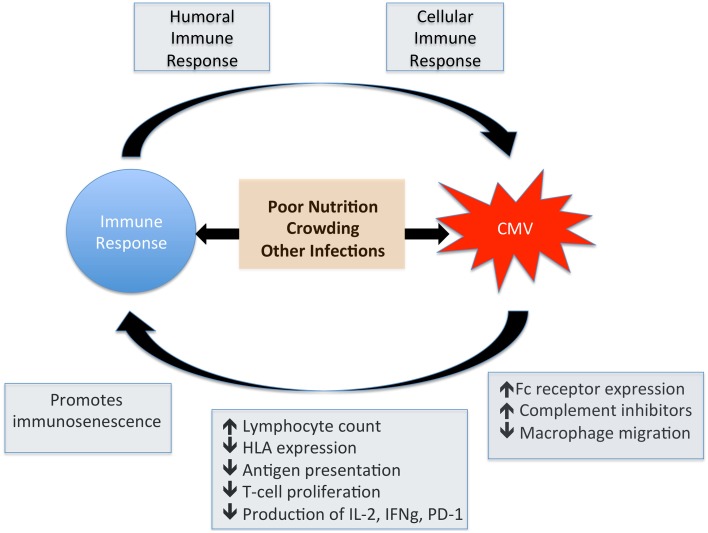
**Impact of CMV on the immune system**. This figure shows the potential for bi-directional interplay between CMV and the immune response. CMV induces a robust humoral and cellular immune response whilst at the same time has a direct influence on normal immune function. Factors such as poor nutrition and low weight, crowded living conditions and other herpes virus coinfections also have an impact on immune function and are associated with increased CMV seroprevalence.

In the pre-ART era, end-organ disease caused by CMV was the most common presentation of HIV infection, particularly retinitis at absolute CD4+ T cell counts < 50/mm3. These manifestations are now much less frequent as ART successfully preserves CD4 counts, but the more subtle immune dysregulation associated with HIV-CMV coinfection is now recognized as a cause for concern. There is good evidence to support the relationship between CMV infection and increased disease progression and mortality in HIV infection (Table 1). However, it remains unknown whether acquisition or reactivation of CMV infection are markers of the immune dysfunction associated with HIV replication, or whether CMV infection itself is a co-factor that promotes HIV progression. These potential pathways are summarized by Figure 3, highlighting the cross-talk between CMV and the immune system.

**Table 1:** **Association between CMV infection and disease progression and mortality in HIV infection in African children**.

**Author**	**Publication Year**	**Study Location**	**Population Studied**	**Findings**
Vilioen	2015	South Africa	124 HIV-infected mothers and their babies	CMV is associated with increased HIV shedding in breast milk
Gumbo	2014	Zimbabwe	257 ART-naïve HIV-positive infants	79% CMV IgG positive by age 6 weeks. No increase in mortality associated with CMV
Tembo	2015	Zambia	303 pediatric inpatients, age 3 weeks to 2 years	CMV viraemia in 41%, associated with being underweight, HIV-positive, or suspected meningitis
Schaftenaar	2014	South Africa	405 ART-naïve HIV-positive children	CMV IgG in 100%, higher titres associated with lower CD4+ T cell count
Manicklal	2014	South Africa	748 neonates born to HIV-infected mothers	Congenital CMV in 2.9%, associated with maternal CD4 count < 200 cells/mm3
Mwaanza	2014	Zambia	395 neonates	Congenital CMV in 3.8%, maternal HIV associated with increased congenital CMV infection
Hsiao	2013	South Africa	425 HIV exposed infants	CMV viraemia is associated with pneumonia in HIV exposed infants
Zampoli	2011	South Africa	202 children with suspected PCP	CMV associated pneumonia more common in HIV infected children
Goussard	2010	South Africa	25 HIV-positive children with suspected PJP	CMV most likely cause of pneumonia and is associated with low CD4 counts and mortality
Slyker	2009	Kenya	64 infants born to HIV-positive mothers	Maternal CMV DNAemia is a significant factor for mortality in HIV infected infants
Roxby	2014	Kenya	141 infants born to HIV-positive mothers	66% acquired CMV by 1 year of age
Slyker	2012	Kenya	474 infants born to HIV-positive mothers	CMV induced T cell activation contributes to rapid disease progression in coinfected infants

*The studies summarized are conducted solely in African children and published between 2009 and 2015*.

In Western settings, active CMV disease in HIV-infected patients has significantly declined in the ART era (Chakravarti et al., 2010), perhaps providing false reassurance that CMV is not a relevant co-pathogen. However, in resource poor settings, including many parts of sub-Saharan Africa, there are few data to inform our understanding of clinical interplay between CMV and HIV. Furthermore, there is increasing evidence to suggest that CMV remains a relevant co-factor in disease progression in individuals with HIV, irrespective of ART (Riou et al., 2015), highlighted by the following three points:

### Accelerated rate of progression of HIV

Progression to an AIDS-defining event has been significantly and independently associated not only with HIV RNA viral load and CD4+ T cell count, but also with CMV DNA (Erice et al., 2003; Fielding et al., 2011). In a population of HIV-infected hemophiliacs, coinfection with CMV adversely influenced the course of the disease; in particular, the age-adjusted relative risk of developing AIDS in CMV-seropositive patients was 2.5 times that in CMV seronegatives (Webster et al., 1989). Conversely, ART dramatically reduces HIV replication, so delaying the development of immunocompromise and disease. Immune reconstitution secondary to ART results in a significant and progressive decline in blood CMV viraemia even in the absence of specific anti-CMV therapy (Connick, 2001).

### Mortality

In studies conducted in developed countries, detectable CMV DNA in plasma (Wohl et al., 2005) or in whole blood (Deayton et al., 2004; Reus et al., 2004) have been shown to be independent predictors of death even after adjusting for HIV RNA level or CD4+ T cell count. Strikingly, a recent study of HIV-positive adults in rural Tanzania that used dried blood spots to detect CMV viraemia found a hazard ratio of 5 for mortality in the presence of CMV (Brantsæter et al., 2012).

A recent large (*n* > 6,000) study of HIV infected individuals in Italy, with high CMV seroprevalence (83.8%), demonstrated an association between CMV IgG and time to a severe non-AIDS event such as the incidence of cardio/cerebro-vascular disease or non-AIDS related death (Lichtner et al., 2015). This study importantly demonstrates the potential for an enhanced disease burden associated with CMV in HIV-infected individuals, in whom coinfection does not necessarily result in AIDS-defining events or well-recognized CMV end-organ disease such as retinitis, but instead a more subtle interplay leads to pathology resulting from systemic inflammation and immune dysregulation. Similarly, other studies report an influence of CMV in driving early aging (e.g., decline in physical function) in HIV-infected adults (Erlandson et al., 2015).

### Pediatric HIV/CMV coinfection

As CMV presents a significant risk factor for HIV disease progression, its impact is likely to be particularly important in children in settings where both viruses are commonly acquired in infancy; several studies document the relationship between CMV coinfection and accelerated HIV progression and increased mortality in children (Kovacs et al., 1999; Goussard et al., 2010; Schaftenaar et al., 2014). In Kenya, detection of maternal CMV DNA in the blood near the time of delivery was associated with three to four-fold increased mortality in HIV-infected infants (Slyker et al., 2009). Strikingly, this relationship remained significant even after controlling for other strong predictors of infant mortality including maternal CD4+ T cell count, infant CD4+ T cell percentage, HIV RNA viral load and maternal death. Studies in HIV infected infants and HIV exposed uninfected infants also link early CMV acquisition with growth delay and cognitive impairment (Roxby et al., 2014).

CD8+ T cell activation has been shown to accompany acute CMV infection in healthy Gambian infants (Miles et al., 2007); since this activation is a strong predictor of HIV disease progression, the acquisition of CMV during primary HIV infection may accelerate infant HIV progression. Again, there is evidence for a reciprocal impact of HIV on CMV, whereby HIV is associated with altered kinetics of CMV replication; specifically, CMV levels peaked at higher levels and tended to decline more slowly in HIV-infected infants (Slyker et al., 2009). Persistent detection of CMV DNA is common in both HIV infected and HEU infants in the 7–12 month post-infection period, suggesting incomplete containment of CMV replication in both these groups of children.

Taken together, these studies suggest that CMV remains a potentially significant pathogen, independently associated with worse outcomes in individuals with HIV, even in the setting of ART. Insight into the epidemiology, pathogenesis, and clinical impact of CMV is currently a neglected component of our understanding of the AIDS epidemic. Crucially, developing insights into the role of CMV in HIV disease could allow interventions including vaccination or anti-viral therapy for at-risk individuals.

As ART becomes more widely accessible to this population for both PMTCT and treatment, it will be important to determine the impact of maternal and infant ART on CMV epidemiology and pathogenesis.

## Treatment and prevention of CMV

CMV disease responds to treatment with a variety of anti-viral agents including ganciclovir, valganciclovir, cidofovir, and foscarnet. Access to appropriate diagnosis followed by anti-viral therapy is difficult in resource-constrained settings, although there is some precedent for CMV therapy in patients with CMV viraemia >1000 copies/ml (Mayaphi et al., 2014), and therapy is clearly advocated for those in whom invasive end-organ disease is suspected or confirmed.

As access to both diagnosis and therapy for CMV disease may be restricted in resource-limited settings, a preventive vaccine is the most robust future strategy to reduce CMV-related morbidity and mortality. Two strategies have been employed to develop CMV vaccines. The first vaccine is live attenuated Towne virus; this has shown weak protection in a human challenge study (Plotkin et al., 1989) but failed to provide protection against infection in CMV seronegative women of childbearing age in an efficacy study (Adler et al., 1995). The second approach is an antigen based approach using recombinant gB/MF59 which showed more promise with ~50% efficacy against CMV infection in a Phase II trial in seronegative women (Pass et al., 2009) and ~50% efficacy in controlling viraemia in seronegative recipients of sero-positive organ transplants (“R-D+”) (Griffiths et al., 2011).

Notably, vaccine trials have only been conducted in the western world but these have resulted in limited success (Wang and Fu, 2014). An effective CMV vaccine for prevention of congenital CMV infection is an increasing priority for the West (Arvin et al., 2004), but the effects of a vaccine could also be far-reaching in developing countries, particularly in populations coinfected with HIV.

## Conclusions

A high incidence of coinfection occurring early in life, impaired CMV containment, persistent CMV DNA detection and a correlation between CMV and HIV peak viral loads suggest that CMV plays an important role in HIV in sub-Saharan Africa, as well as in other resource poor settings. Despite compelling evidence that these infections impact negatively on one another, this field is relatively neglected, particularly in some of the world's most vulnerable populations including neonates and developing world settings.

Although florid end-organ CMV disease may no longer be a substantial problem due to the increasing success of ART, there is growing evidence that its impact as a co-pathogen remains highly relevant. This may be mediated by immune activation and systemic inflammation driven by CMV, increasing the risks of HIV-related morbidity and mortality. There are also interesting data suggesting an effect of CMV on immune ontogeny, suggesting it has a role in determining the nature and efficacy of immune responses to a variety of other infections. This effect may be particularly pertinent in children.

Significant therapeutic benefits could potentially be derived from interventions to reduce the incidence of CMV infection, or to reduce the sequelae of chronic infection including disease reactivation and long-term immune activation.

## Funding

PCM has salary support from the NIHR, and research grants from Oxford University Clinical Academic Graduate School (OUCAGS) and The John Fell Fund. PG is funded by the Wellcome Trust (WT104748MA) and PK is also funded by the Wellcome Trust (WT091663MA).

### Conflict of interest statement

The authors declare that the research was conducted in the absence of any commercial or financial relationships that could be construed as a potential conflict of interest.

## Abbreviations

HIV: Human Immunodeficiency Virus (denotes HIV-1)
CMV: Cytomegalovirus
HHV5: Human Herpes Virus-5
ART: Antiretroviral Therapy
MSM: Men who have Sex with Men
HEU: HIV Exposed Uninfected
AICD: Activation Induced Cell Death
PMTCT: Prevention of Mother-To-Child Transmission.
